# Comparison of vasodilators for heart failure: a systematic review and network meta-analysis of RCTs

**DOI:** 10.1186/s12872-026-06278-7

**Published:** 2026-07-11

**Authors:** Qin Zhao, Shanmei Liu, Yichang Zhou, Jianquan Zhao

**Affiliations:** 1 Department of cardiovascular medicine, Bayannur Hospital, Bayannur, Inner Mongolia 015000 China; 2 Dalian Medical University, Laoning 116000 Dalian, China

**Keywords:** Heart Failure, Vasodilator, Safety, Network meta-analysis, Systematic review

## Abstract

**Background:**

The effect of vasodilators in the management of heart failure (HF) remains inconclusive. This study aims to evaluate and compare the overall benefits of vasodilators to patients with HF.

**Methods:**

PubMed, Embase, Cochrane Library, and Web of Science were searched from database inception to December 27th, 2021. Randomized controlled trials (RCTs) assessing the efficacy of vasodilators in the treatment of HF were included. The Cochrane risk of bias assessment tool was applied to assess the risk of bias of included studies. Meta-analysis was performed using R and Stata 15.0 software.

**Results:**

A total of 5517 articles were retrieved, and 18 eligible RCTs were finally included, with 9188 participants. Network meta-analysis showed no difference in the mortality, readmission rate, incidence of adverse cardiovascular events, and serum creatinine (Scr) among different vasodilators. Urapidil was more effective in reducing NT-pro-BNP, compared with nitroglycerin [WMD=-975.64, 95%CI (-1558.32, -320.14)].

**Conclusion:**

There are no differences in the efficacy and safety among vasodilators in the management of HF. Especially, although nitroprusside may be the most effective in reducing mortality, there is no significant difference between other drugs. Given the low certainty of the evidence, current evidence suggests that vasodilators have limited efficacy in the routine management of HF. More well-designed RCTs are needed to validate these findings.

**Trial registration:**

PROSPERO (CRD42022351231).

**Supplementary Information:**

The online version contains supplementary material available at https://doi.org/10.1186/s12872-026-06278-7.

## Introduction

Heart failure (HF) refers to a complex clinical syndrome induced by a structural or functional disorder of ventricular filling or ejection. It is a chronic stage of multiple cardiac diseases, with an increasing incidence rate around the world [1]. HF is typically classified into HFrEF, HFmrEF, and HFpEF, according to the left ventricular ejection fraction (LVEF), or chronic HF (CHF) and acute HF (AHF) based on the episode of the disease. The New York Heart Association (NYHA) classifies HF according to the severity of the symptoms [2]. The global incidence of HF is rising rapidly, with more than 64 million diagnosed cases. It is globally prevalent, and the number of HF patients may dramatically increase with its ever-changing demographic and epidemiological characteristics [3]. The incidence is usually estimated as 1000 person-years or 1–20 cases per 1000 people (varying across regions and populations). Most of the data come from Europe and North America, with an incidence of approximately 2–3 cases per 1000 people. The incidence rises with aging [4]. The incidence of HF in developed countries may be decreasing, while the overall incidence is increasing due to population aging and life span extension attributed to advances in medical technology [5, 6]. As the study on HF treatment deepens, multiple pharmacologic agents have been developed and used to treat different types of HF and its complications. For example, diuretics are recommended for HF patients with fluid retention to alleviate their symptoms. Angiotensin-converting enzyme inhibitor (ACEI), angiotensin receptor blocker (ARB), and angiotensin receptor enkephalin enzyme inhibitor (ARNI) can decrease the incidence and mortality of HF by regulating renin-angiotensin system (RAS). Beta adrenergic receptor-blocker (BB) can reduce the overall risk of mortality and hospital admission. Vasodilators can rapidly attenuate pulmonary edema [1, 7]. Vasodilators play a key role in the treatment of HF, especially AHF, and can be used in combination therapy for patients concomitant with angina or hypertension. Both the AHA and ECS guidelines recommend hydralazine combined with isosorbide dinitrate (2b) for patients with current or history of symptomatic HFrEF who cannot be given first-line drugs, such as ARNI, ACEI, or ARB due to drug intolerance or renal insufficiency, in order to reduce the incidence and mortality. In the treatment of acute HF, vasodilators (2b), such as intravenous nitroglycerin or sodium nitroprusside, are recommended to reduce congestion [8, 9]. It acts by dilating both arteries and veins to lighten cardiac preload and afterload so as to improve the symptoms [10]. However, the safety of vasodilators remains controversial. For instance, nitrates alleviate cardiac filling pressure and systolic pressure by reducing peripheral resistance, which is often accompanied by side effects like headache and reflex tachycardia. These side effects might occur within 24 h after patients receive intravenous nitroglycerin, and drug resistance could occur in about 20% of the patients [11, 12]. Sodium nitroprusside could cause postural hypotension [13]. Urapidil has double effects. It induces peripheral vessels and pulmonary artery dilation by binding with peripheral α-1 adrenergic receptors and central 1 A serotonergic (5-HT-1 A) receptors [14], while it also causes adverse reactions due to its rapid antihypertensive effect. The safety of urapidil in the treatment of HF remains unclear, in that few relevant clinical trials have been conducted. The rhBNP dilates body and pulmonary vessels while promoting the excretion of urinary sodium, producing a diuretic effect, and inhibits the renin-angiotensin-aldosterone system (RAAS) [15]. However, it may increase the risk of cardiovascular adverse events [16].

The efficacy of vasodilators in HF treatment remains controversial and is not systematically assessed. There are few data available regarding various types of vasodilators due to the lack of head-to-head studies. Network meta-analysis (NMA) can directly and indirectly compare multiple interventions. Therefore, we conduct this NMA to assess the efficacy and safety of vasodilators in the treatment of HF.

## Methods

This NMA was conducted in strict accordance with the *Cochrane Handbook for the Systematic Review of Interventions and the Preferred Reporting Items for Systematic Review and Meta-Analyses* guideline (http://training.cochrane.org/handbook) [17]. The study protocol was registered on PROSPERO (registration No. CRD42022351231).

### Search strategy

PubMed, EMBASE, Cochrane Library, and Web of Science were searched from database inception to December 17th 2021 for randomized controlled trials (RCTs) on vasodilators in the treatment of HF. Search strategies were designed based on the combination of medical subject headings and free words. Reference lists of retrieved studies were also searched for potentially eligible studies. We performed a second search in each database after data extraction to further confirm that no eligible study was missed. Detailed search strategies of PubMed are presented in Table S1.

### Inclusion and exclusion criteria

#### Inclusion criteria

1. RCTs with over 10 participants.
2. Participants recruited were diagnosed with HF of any type, and were aged over 18.
3. Studies compared vasodilators with placebo or other counterparts. Vasodilators included nitroglycerin, isosorbide mononitrate (ISDN), sodium nitroprusside, urapidil, and rhBNP.
4. Outcomes of interest were reported, such as mortality, readmission rate, incidence of cardiovascular adverse events (Includes: all-cause death, non-fatal myocardial infarction, malignant arrhythmia, or re-hospitalization due to worsening heart failure), serum creatinine (SCr), ejection fraction (EF), and NT-pro-BNP.
5. Studies reported and published in English.

#### Exclusion criteria

1. Full texts unavailable.
2. Studies published in non-English languages.
3. Repeated publication, animal study, case report, comment, meta-analysis, letter, conference summary, etc.
4. Studies applied other conventional treatments as control.
5. Studies applied other conventional agents as interventions for HF.
6. Outcome measures unavailable or unextractable.

### Data extraction

Titles and abstracts of retrieved articles were screened by two reviewers (QZ and SL) independently to remove irrelevant studies. The full texts of the remaining articles were read to determine eligible studies. Disagreements were settled via discussion with a third reviewer (JZ).

Two reviewers (QZ and SL) independently extracted the data using a standardized form. The extracted data mainly included the name of the first author, publication date, characteristics of participants, sample size, mean age, specific interventions, outcome measures, and follow-up duration. Data extraction results were cross-checked by the two reviewers, and any discrepancies were settled through discussion with a third reviewer (JZ).

### Quality assessment

Quality assessment of included studies was conducted by two reviewers (QZ and SL) independently using Cochrane Risk of Bias Assessment Tool [18]. This tool contained 7 domains: randomization sequence generation (selection bias), allocation concealment (selection bias), blinding of participants and personnel (performance bias), blinding of outcome assessment (detection bias), incomplete outcome data (attrition bias), selective reporting (reporting bias), and other bias. The included studies were graded as “low risk”, “unclear risk”, or “high risk”. Disagreements were settled via discussion with the third reviewer (JZ). We assessed the certainty of evidence for each outcome by considering RoB, imprecision, indirectness, inconsistency, publication bias, intransitivity, incoherence, and factors for up-rating using the Grading of Recommendations Assessment, Development and Evaluation (GRADE) approach and extension for network meta-analysis [19–21]. 

### Data analysis

Dichotomous data were expressed as risk ratio (RR), and continuous data were expressed as mean difference (MD), with 95% confidence interval (95%CI). Given the heterogeneity among included studies, Bayesian stratified random-effect model was applied for multiple comparisons of different vasodilator regimens in HF treatment. The model was built via Markov chain Monte Carlo (MCMC) method. Four Markov chains run simultaneously, and the frequency of simulated annealing was set as 20,000, and modeling was completed after 50,000 simulation iterations. Deviation information standard (DIC) was adopted to evaluate the model fitting and global consistency. The node-splitting method was adopted to test the local consistency, if a closed loop was observed in network diagrams. Additionally, interventions were ranked based on SUCRA, and a league table was provided to compare the differences among interventions. Publication bias was assessed using a funnel plot. All data analyses were performed using Stata 15.1 and R (Ver 4.0.3) software.

## Results

### Study selection

**Fig. 1:**
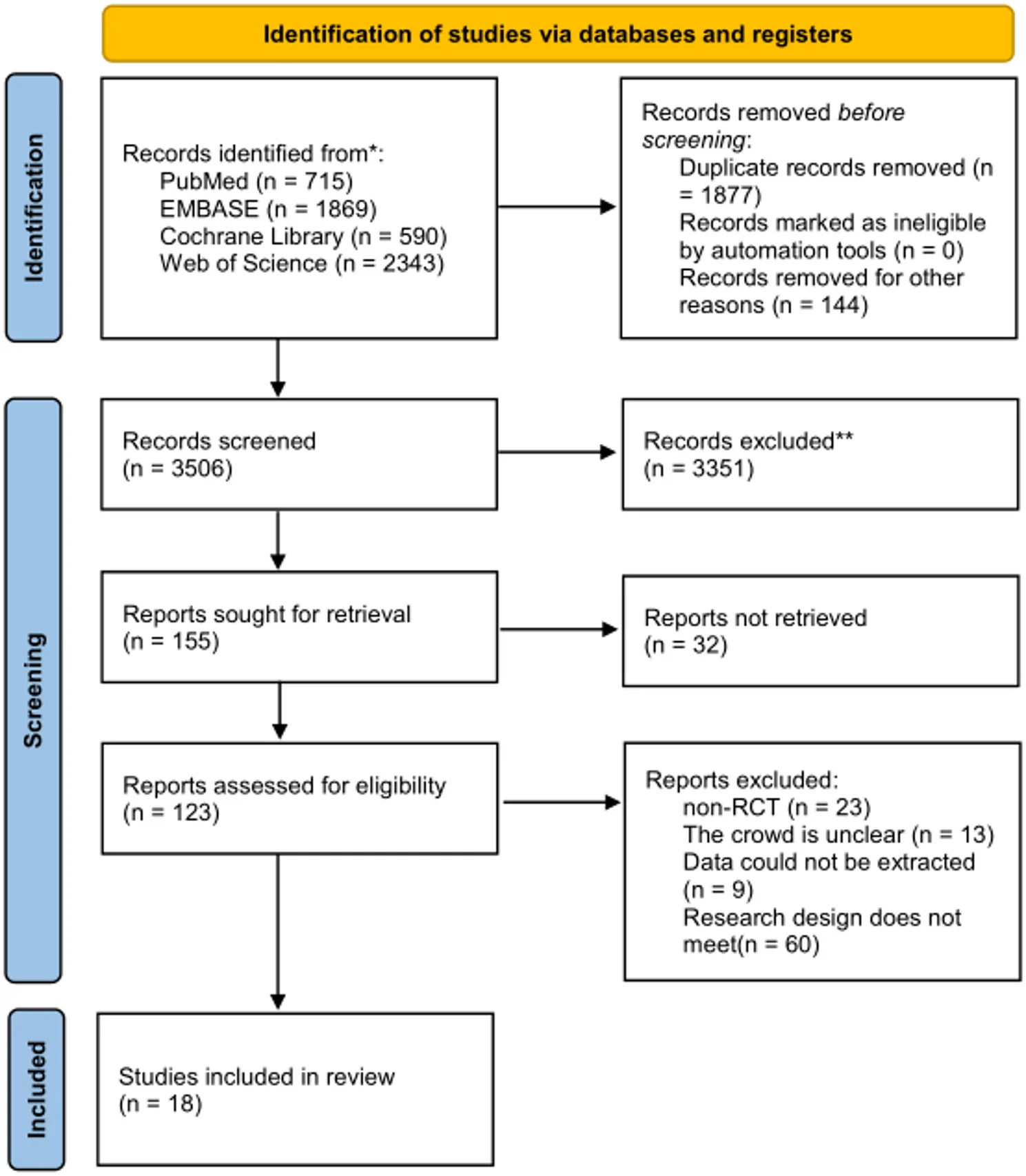
Flowchart of searching and screening for the studies

A total of 5517 articles were retrieved. After duplicate publications were removed, 3506 studies remained. We removed 3351 irrelevant studies after browsing titles and abstracts, and excluded 137 ineligible studies after reading full texts. Finally, 18 RCTs were included. The study selection process is shown in Fig. 1.

### Characteristics of included studies

**Fig. 2:**
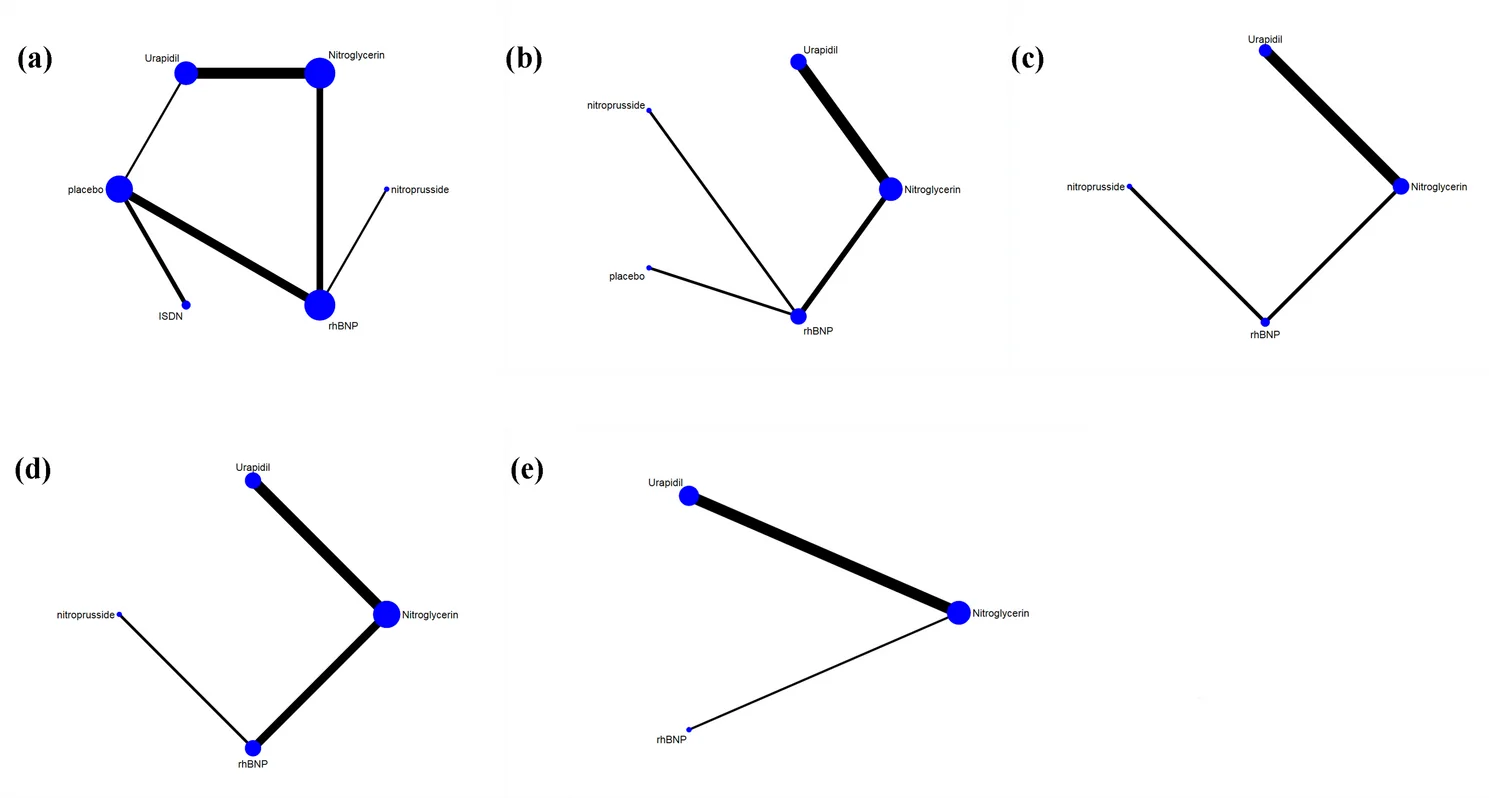
Network plot of available comparisons between different treatments in patients with heart failure (HF). The nodes’ size is proportional to number of subjects allocated ineach treatment. The thickness of continuous lines is proportional to the number of studies directly comparing the two treatments. The endpoints considered are: (**a**) mortality, (**b**) re-admission rate, (**c**) cardiovascular adverse events, (**d**) Scr, (**e**) NT-pro-BNP

A total of 18 RCTs [22–39] were included in this NMA, with a total of 9188 HF patients. Among the included studies, 9 were multi-center trials, and the rest were single-center studies. The follow-up duration ranged from 1 to 6 months. Characteristics of included studies are presented in Table S2, and the network diagram of each intervention is shown in Fig. 2.

### Quality assessment

The quality of included studies was assessed using the Cochrane risk of bias assessment tool. Among the 18 studies, nine studies described randomization sequence generation process, such as computer-generated random number or random number table. Four studies reported proper allocation concealment, whereas the other 14 studies failed to report the process or applied improper allocation concealment. Four studies reported the lack of double-blinding, three studies failed to report the blinding implementation, and another four studies failed to perform blinding of assessment. Three studies were graded as high risk of bias due to incomplete data. The other sources of bias were unclear due to the insufficient information in all included studies. The risk of bias assessment results of included RCTs are shown in Fig. S1.

### Network meta-analysis of included studies

#### Mortality

A total of 16 studies reported the mortality with the final follow-up duration (ranging from 1 month to 6 months among the included studies), involving 5 vasodilators (nitroglycerin, ISDN, sodium nitroprusside, urapidil, and rhBNP). The overall network meta-analysis showed no statistically significant difference between vasodilators and placebo, and no significant difference was observed in the pairwise comparison of these agents (Fig. 2a; Table 1). These results indicated that no vasodilators significantly reduced mortality. The level of evidence of this outcome measure was evaluated as low according to the GRADE system (Table S3).

**Table 1:** Results from the multiple-treatment comparison analyses in the overall analysis

mortality (RR [95% Cl])					
ISDN	0.74 (0.07, 11.28)	1.18 (0.03, 100.27)	0.38 (0.05, 2.49)	0.46 (0.05, 4.75)	0.49 (0.04, 6.94)
	Nitroglycerin	1.57 (0.05, 87.29)	0.52 (0.08, 2)	0.62 (0.15, 2.21)	0.66 (0.12, 2.57)
		nitroprusside	0.32 (0.01, 8.04)	0.4 (0.01, 9.17)	0.41 (0.01, 14.61)
			placebo	1.16 (0.52, 4.26)	1.29 (0.23, 8.25)
				rhBNP	1.07 (0.17, 5.52)
					Urapidil
re-admission rate (RR [95% Cl])					
Nitroglycerin		10.16 (0.72, 460.51)	1.58 (0.27, 10.07)	1.55 (0.37, 7.27)	0.71 (0.32, 1.26)
		nitroprusside	0.16 (0, 1.76)	0.16 (0, 1.37)	0.07 (0, 1.02)
			placebo	0.98 (0.33, 2.87)	0.44 (0.06, 2.68)
				rhBNP	0.45 (0.08, 2.07)
					Urapidil
incidence of cardiovascular adverse events (RR [95% Cl])					
Nitroglycerin		6.81 (0.53, 112.95)		2.93 (0.41, 31.47)	0.43 (0.17, 0.98)
		nitroprusside		0.44 (0.09, 2.12)	0.06 (0, 0.9)
				rhBNP	0.15 (0.01, 1.21)
					Urapidil
Scr (WMD [95% Cl])					
Nitroglycerin		3.62 (-19.87, 27.37)		0.48 (-10.79, 11.99)	0.43 (-8.36, 9.18)
		nitroprusside		-3.13 (-23.86, 17.43)	-3.19 (-28.46, 21.75)
				rhBNP	-0.06 (-14.63, 14.19)
					Urapidil
NT-pro-BNP (WMD [95% Cl])					
Nitroglycerin				-126.51 (-1457.46, 1203.99)	**-975.64 (-1558.32**,** -320.14)**
				rhBNP	-848.4 (-2277.02, 656.78)
					Urapidil

#### Re-admission rate

Eight studies reported the re-admission rate, involving 4 vasodilators (nitroglycerin, sodium nitroprusside, urapidil, and rhBNP). The overall network meta-analysis showed no statistically significant difference between vasodilators and placebo, and no significant difference was observed in the pairwise comparison of these agents (Fig. 2b; Table 1). These results indicated that no vasodilators significantly reduced the re-admission rate. The level of evidence of this outcome measure was evaluated as low according to the GRADE system (Table S3).

#### Incidence of cardiovascular adverse events

Five studies reported the incidence of cardiovascular adverse events, involving 4 vasodilators (nitroglycerin, ISDN, sodium nitroprusside, urapidil, and rhBNP). The overall network meta-analysis showed no statistically significant difference between vasodilators and placebo. No significant difference was observed in the pairwise comparison of these agents. These agents presented to be of similar safety (Fig. 2c; Table 1). The level of evidence of this outcome measure was evaluated as low according to the GRADE system (Table S3).

#### SCr

Eight studies reported SCr, involving 4 vasodilators (nitroglycerin, sodium nitroprusside, urapidil, and rhBNP). The overall network meta-analysis showed no significant difference, and no significant difference was observed in the pairwise comparison of these agents, indicating that no vasodilators significantly reduced SCr (Fig. 2d; Table 1). The level of evidence of this outcome measure was evaluated as low according to the GRADE system (Table S3).

#### NT-pro‐BNP

Six studies reported NT-pro-BNP, involving 3 vasodilators (nitroglycerin, urapidil, and rhBNP). The overall network meta-analysis showed that urapidil was more effective in reducing NT-pro-BNP, compared with nitroglycerin [WMD=-975.64, 95%CI (-1558.32, -320.14)] (Fig. 2e; Table 1). The level of evidence of this outcome measure was evaluated as low according to the GRADE system (Table S3).

### Ranking

**Fig. 3:**
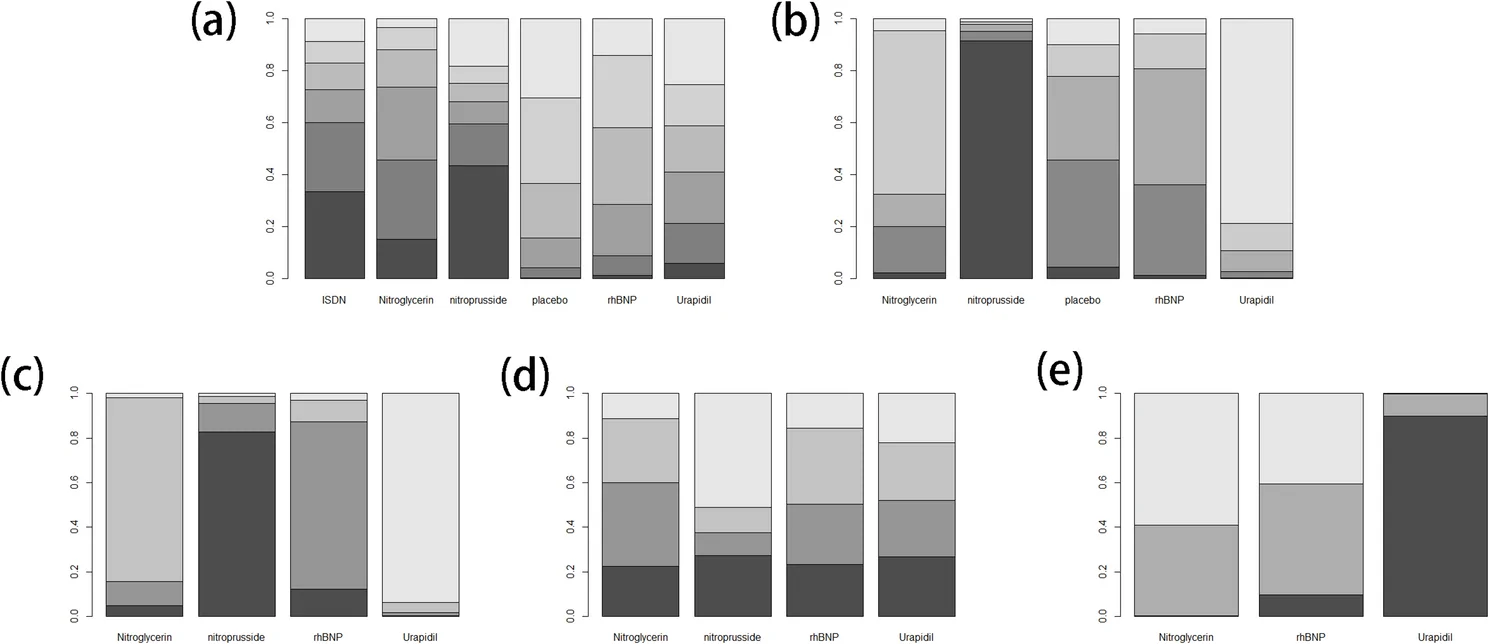
Rank probabilities based on the network meta-analysis. **a** mortality, (**b**) re-admission rate, (**c**) cardiovascular adverse events, (**d**) Scr, (**e**) NT-pro-BNP. Notes: A darker color represents a higher ranking. A higher height among the blocks with the same color represents a higher probability. That is, under the same indicator, the intervention with the darkest color and the highest height has the greatest probability of ranking first

The probability ranking chart showed that nitroprusside ranked the top in re-admission rate (95.6%) and cardiovascular adverse events (92.2%). In mortality and SCr, there was little difference among the vasodilators. Urapidil (94.8%) ranked the top in NT-pro-BNP (shown in Fig. 3; Table 2). Although nitroprusside showed the highest SUCRA value for readmission rate (95.6%), no statistically significant differences were observed between nitroprusside and other vasodilators in pairwise comparisons (Table 1). This suggested that its high SUCRA ranking may not translate into a clinically meaningful advantage.

**Table 2:** Results of surface under cumulative ranking curve (SUCRA)

vasodilators	mortality	re-admission rate	cardiovascular adverse events	Scr	NT-pro-BNP
SUCRA (%)					
ISDN	68.4	-	-	-	-
Nitroglycerin	62.7	37.4	39.6	56.9	20.6
nitroprusside	66.2	**95.6**	**92.2**	38.1	-
rhBNP	36.3	52.4	65.5	52.8	34.7
Urapidil	40.4	10.9	2.7	52.2	**94.8**
placebo	26	53.8	-	-	-

### Publication bias and inconsistency

Publication bias was assessed using a funnel plot constructed by Stata. Different colors represented comparisons of different interventions. As for mortality, re-admission rate, incidence of cardiovascular adverse events, sCr, EF, and NT-pro-BNP, funnel plots of four outcomes showed no significant publication bias (shown in Fig. S2). The nonnegative matrix analysis failed to contain the inconsistency.

## Discussion

In this systematic review and network meta-analysis, we reviewed direct and indirect evidence from 19 RCTs (Fig. 1), and compared the efficacy and safety of different vasodilators in the treatment of HF. Most of the interventions lack direct comparisons, so indirect comparisons have played a critical role in our study. To our knowledge, this is the first nonnegative matrix analysis to compare the effect of vasodilators on mortality, re-admission rate, and renal prognosis in patients with HF. As shown in Table 1, urapidil was significantly effective in improving NT-pro-BNP, with statistical significance. Moreover, pair comparisons of the 5 vasodilators showed no statistically significant difference in mortality, re-admission rate, SCr, and cardiovascular adverse events. Nonetheless, the probability ranking indicated that nitroprusside was statistically effective in reducing re-admission rates and cardiovascular adverse events (Table 2). However, a statistically significant indicator might not be of clinical significance. In fact, current evidence is insufficient to support the routine use of vasodilators in the management of HF, and a single vasodilator cannot effectively reduce the incidence of poor outcomes in HF patients. The efficacy and safety are similar across the vasodilators.

HF is not a separate symptom, but a clinical syndrome consisting of multiple symptoms (such as dyspnea, ankle edema, fatigue) and signs (such as increased jugular vein pressure, inspiratory moist crackles, peripheral edema) [13]. Treatment of HF is based on different concomitant symptoms and clinical manifestations. For instance, diuretics for fluid retention is used to alleviate the symptom, while ACEI, ARB, and BB are effective for decreasing blood pressure and enhancing myocardial contraction [40]. Vasodilators play a critical role in the treatment of HF, especially in attenuating pulmonary edema of AHF. Nitroglycerin, isosorbide mononitrate, sodium nitroprusside, urapidil, and rhBNP are commonly-used vasodilators, among which nitroglycerin and sodium nitroprusside are more preferred options [10].

A previous meta-analysis by Shi et al. [41], has revealed that urapidil is more effective in the N-terminal prohormone of BNP compared with nitroglycerin, while urapidil results in poorer SCr (urapidil-induced hepatorenal injury is milder than that caused by nitroglycerin). Our findings confirmed that urapidil was superior to nitroglycerin in the N-terminal prohormone of BNP, and the difference was statistically significant. However, there was no statistical difference in SCr between the two groups. The reason for such a discrepancy is that the study by Shi et al. measures the outcomes after 7-day treatment, whereas our study focuses on the outcomes after 2-day treatment. In a study by Wang et al. [34], the nitroglycerin group has increased SCr after 2–7 days of treatment. Given this situation, we added the changes after 2 days of treatment. However, the results need to be further validated by more trials. Zhang et al. [42] suggest no significant difference between rhBNP and NIT in reducing mortality and re-admission rate. Another study by Gong et al. [16] indicates that nesiritide does not increase the overall mortality risk compared with placebo. Their findings are consistent with ours. On the other hand, the study by Gong et al. [16] suggests that rhBNP is associated with an increased risk of cardiovascular adverse events compared with the control group. However, our study revealed that rhBNP regimen failed to increase the risk of cardiovascular adverse events compared with placebo. The reason for the inconsistent findings may be that our study mainly focused on major adverse cardiovascular events (MACE) instead of bradycardia or hypotension.

Most of the current meta-analyses focus on the comparison of one or two vasodilators in HF treatment, such as rhBNP, urapidil, and nitroglycerin. Their conclusions vary in terms of mortality, adverse reactions, renal function, and cardiovascular adverse events. Some studies have yielded positive results, whereas some report negative results. Our study, for the first time, reviewed and assessed five types of vasodilators, and integrated direct and indirect pair comparisons. However, our results showed no difference in the mortality, readmission rate, incidence of adverse cardiovascular events, and SCr among different vasodilators. Urapidil was found to be more effective in reducing NT-pro-BNP, compared with nitroglycerin. These findings are similar to those of previous reviews.

This study has some strengths and limitations. As for strengths, this is the first network meta-analysis comparing five types of vasodilators. This study has assessed the robustness of evidence from current RCTs, and has filled the gap in systematic review and meta-analysis on some vasodilators. Meanwhile, the efficacy and safety of these vasodilative agents are compared directly and indirectly. However, some limitations should be acknowledged. Firstly, few RCTs are included, and the data from some included studies cannot be obtained. Secondly, most included studies mainly focus on one or two vasodilators. Therefore, it is infeasible to compare all five vasodilators in most of the outcome measures. Thirdly, due to the incomplete reporting of patient information in the original studies, especially the limited description of heart failure classification and staging, subgroup analysis is not conducted based on different populations. Fourthly, most included studies are conducted in China and the United States. Regional and racial bias is unavoidable. Lastly, the clinical relevance of the observed ranking differences appears limited, as no single agent shows a clear advantage in reducing hard endpoints like mortality. Therefore, this result should be interpreted with caution.

Therefore, more high-quality RCTs are needed to further confirm and improve our findings. Future studies are desired to involve more and clearer patient information, which can facilitate further research on treatments for patients with specific subtypes or different stages of HF. Secondly, more studies are warranted to further explore the administration regimens of vasodilators (such as dosage, administration time, and administration route). Finally, future studies should measure drug effects and safety over a consistent time frame and conduct long-term follow-ups.

## Conclusion

The efficacy and safety of vasodilators in HF treatment need to be further validated. Current evidence does not support the routine use of vasodilators in the management of HF, and there are no significant differences between vasodilators. Moreover, it is important to note that the overall quality of evidence for all outcomes is rated as low according to GRADE, primarily due to imprecision and risk of bias. More relevant studies are expected to produce more robust evidence, especially for some agents that are less studied.

## Supplementary Information

Supplementary Material 1: Table S1 Search strategy in Pubmed. Table S2 Characteristics of the included studies. Table S3: GRADE assessment. Fig. S1 Risk of bias graph. Fig. S2 Funnel plots of outcomes (a) Mortality, (b) re-admission rate, (c) incidence of cardiovascular adverse events, (d) sCr, (e) NT-pro-BNP.

## Data Availability

The datasets used and/or analysed during the current study are available from the corresponding author on reasonable request.

## Abbreviations

RCTs: Randomized controlled trials
HF: Heart failure
LVEF: Left Ventricular Ejection Fraction
CHF: chronic HF
AHF: acute HF
NYHA: New York Heart Association
ACEI: angiotensin-converting enzyme inhibitor
ARB: angiotensin receptor blocker
ARNI: angiotensin receptor enkephalin enzymes inhibitor
RAS: renin-angiotensin system
BB: Beta adrenergic receptor-blocker
RAAS: Renin-Angiotensin-Aldosterone System
NMA: Network meta-analysis
SCr: serum creatinine
EF: ejection fraction
MD: mean difference
MCMC: Markov chain Monte Carlo
DIC: Deviation information standard
