# Supplementary material for: Comparison of vasodilators for heart failure: a systematic review and network meta-analysis of RCTs
Source: BMC Cardiovasc Disord. 2026 Jul 11;26:878. doi: 10.1186/s12872-026-06278-7 (PMC13640276; doi:10.1186/s12872-026-06278-7)
Supplement: Supplementary file 1 [file 12872_2026_6278_MOESM1_ESM.docx]

**Additional file 1**

**Table S1** Search strategy in Pubmed

| Search number | Query | Results |
| --- | --- | --- |
| 16 | ((((((((Heart Failure[Title/Abstract]) OR (Cardiac Failure[Title/Abstract])) OR (Heart Decompensation[Title/Abstract])) OR (Decompensation, Heart[Title/Abstract])) OR (Myocardial Failure[Title/Abstract]))) OR ("Heart Failure"[Mesh])) AND ((((((((((((((((((("Isosorbide Dinitrate"[Mesh]) OR (Isosorbide Dinitrate[Title/Abstract])) OR (Dinitrate, Isosorbide[Title/Abstract])) OR (Dilatrate[Title/Abstract])) OR (Iso Bid[Title/Abstract])) OR (Isodinit[Title/Abstract])) OR (Isoket[Title/Abstract])) OR (Sorbonit[Title/Abstract])) OR (Isomak R[Title/Abstract])) OR (Isordil[Title/Abstract])) OR (Isotrate[Title/Abstract])) OR (Nitrosorbide[Title/Abstract])) OR (Sorbitrate[Title/Abstract])) OR (Cardonit 40[Title/Abstract])) OR (Isoket Retard 120[Title/Abstract])) OR (((((((((((((((((((((((((((("Nitroglycerin"[Mesh]) OR (Nitroglycerin[Title/Abstract])) OR (Glyceryl Trinitrate[Title/Abstract])) OR (Trinitrate, Glyceryl[Title/Abstract])) OR (Nitrolan[Title/Abstract])) OR (Nitrospan[Title/Abstract])) OR (Nitrostat[Title/Abstract])) OR (Perlinganit[Title/Abstract])) OR (Nitrong[Title/Abstract])) OR (Susadrin[Title/Abstract])) OR (Sustac[Title/Abstract])) OR (Sustak[Title/Abstract])) OR (Sustonit[Title/Abstract])) OR (Transderm Nitro[Title/Abstract])) OR (Nitroglyn[Title/Abstract])) OR (Trinitrin[Title/Abstract])) OR (Trinitrolong[Title/Abstract])) OR (Anginine[Title/Abstract])) OR (Dynamite[Title/Abstract])) OR (Gilustenon[Title/Abstract])) OR (Nitrangin[Title/Abstract])) OR (Nitro Bid[Title/Abstract])) OR (Nitro Dur[Title/Abstract])) OR (Nitrocard[Title/Abstract])) OR (Nitroderm[Title/Abstract])) OR (Nitroderm TTS[Title/Abstract])) OR (Nitrol[Title/Abstract])) OR (Tridil[Title/Abstract]))) OR (((((((((((((((("Nitroprusside"[Mesh]) OR (Nitroprusside[Title/Abstract])) OR (Cyanonitrosylferrate[Title/Abstract])) OR (Nitroferricyanide[Title/Abstract])) OR (Nipruton[Title/Abstract])) OR (Nitriate[Title/Abstract])) OR (Nitropress[Title/Abstract])) OR (Nitroprusside, Disodium Salt, Dihydrate[Title/Abstract])) OR (Nipride[Title/Abstract])) OR (Naniprus[Title/Abstract])) OR (Nitroprussiat Fides[Title/Abstract])) OR (Sodium Nitroprusside[Title/Abstract])) OR (Nitroprusside, Sodium[Title/Abstract])) OR (Nitroprusside, Disodium Salt[Title/Abstract])) OR (Disodium Salt Nitroprusside[Title/Abstract])) OR (Ketostix[Title/Abstract]))) OR ((((((Urapidil[Title/Abstract]) OR (6-(3-(4-(o-methoxyphenyl)-1-piperazinyl)propylamino)-1,3-dimethyluracil[Title/Abstract])) OR (urapidil monohydrochloride[Title/Abstract])) OR (urapidil, 5-(14)C-labeled[Title/Abstract])) OR (Ebrantil[Title/Abstract])) OR (urapidil, 2,3-(14)C2-labeled[Title/Abstract]))) OR ((Recombinant Human Brain Natriuretic Peptide[Title/Abstract]) OR (rhBNP[Title/Abstract])))) AND ((((((random[Title/Abstract]) OR (randomized[Title/Abstract])) OR (placebo[Title/Abstract])) OR (trail*[Title/Abstract])) OR (control[Title/Abstract])) OR (controlled[Title/Abstract])) | 715 |
| 15 | (((((random[Title/Abstract]) OR (randomized[Title/Abstract])) OR (placebo[Title/Abstract])) OR (trail*[Title/Abstract])) OR (control[Title/Abstract])) OR (controlled[Title/Abstract]) | 4,038,883 |
| 14 | (((((((Heart Failure[Title/Abstract]) OR (Cardiac Failure[Title/Abstract])) OR (Heart Decompensation[Title/Abstract])) OR (Decompensation, Heart[Title/Abstract])) OR (Myocardial Failure[Title/Abstract]))) OR ("Heart Failure"[Mesh])) AND ((((((((((((((((((("Isosorbide Dinitrate"[Mesh]) OR (Isosorbide Dinitrate[Title/Abstract])) OR (Dinitrate, Isosorbide[Title/Abstract])) OR (Dilatrate[Title/Abstract])) OR (Iso Bid[Title/Abstract])) OR (Isodinit[Title/Abstract])) OR (Isoket[Title/Abstract])) OR (Sorbonit[Title/Abstract])) OR (Isomak R[Title/Abstract])) OR (Isordil[Title/Abstract])) OR (Isotrate[Title/Abstract])) OR (Nitrosorbide[Title/Abstract])) OR (Sorbitrate[Title/Abstract])) OR (Cardonit 40[Title/Abstract])) OR (Isoket Retard 120[Title/Abstract])) OR (((((((((((((((((((((((((((("Nitroglycerin"[Mesh]) OR (Nitroglycerin[Title/Abstract])) OR (Glyceryl Trinitrate[Title/Abstract])) OR (Trinitrate, Glyceryl[Title/Abstract])) OR (Nitrolan[Title/Abstract])) OR (Nitrospan[Title/Abstract])) OR (Nitrostat[Title/Abstract])) OR (Perlinganit[Title/Abstract])) OR (Nitrong[Title/Abstract])) OR (Susadrin[Title/Abstract])) OR (Sustac[Title/Abstract])) OR (Sustak[Title/Abstract])) OR (Sustonit[Title/Abstract])) OR (Transderm Nitro[Title/Abstract])) OR (Nitroglyn[Title/Abstract])) OR (Trinitrin[Title/Abstract])) OR (Trinitrolong[Title/Abstract])) OR (Anginine[Title/Abstract])) OR (Dynamite[Title/Abstract])) OR (Gilustenon[Title/Abstract])) OR (Nitrangin[Title/Abstract])) OR (Nitro Bid[Title/Abstract])) OR (Nitro Dur[Title/Abstract])) OR (Nitrocard[Title/Abstract])) OR (Nitroderm[Title/Abstract])) OR (Nitroderm TTS[Title/Abstract])) OR (Nitrol[Title/Abstract])) OR (Tridil[Title/Abstract]))) OR (((((((((((((((("Nitroprusside"[Mesh]) OR (Nitroprusside[Title/Abstract])) OR (Cyanonitrosylferrate[Title/Abstract])) OR (Nitroferricyanide[Title/Abstract])) OR (Nipruton[Title/Abstract])) OR (Nitriate[Title/Abstract])) OR (Nitropress[Title/Abstract])) OR (Nitroprusside, Disodium Salt, Dihydrate[Title/Abstract])) OR (Nipride[Title/Abstract])) OR (Naniprus[Title/Abstract])) OR (Nitroprussiat Fides[Title/Abstract])) OR (Sodium Nitroprusside[Title/Abstract])) OR (Nitroprusside, Sodium[Title/Abstract])) OR (Nitroprusside, Disodium Salt[Title/Abstract])) OR (Disodium Salt Nitroprusside[Title/Abstract])) OR (Ketostix[Title/Abstract]))) OR ((((((Urapidil[Title/Abstract]) OR (6-(3-(4-(o-methoxyphenyl)-1-piperazinyl)propylamino)-1,3-dimethyluracil[Title/Abstract])) OR (urapidil monohydrochloride[Title/Abstract])) OR (urapidil, 5-(14)C-labeled[Title/Abstract])) OR (Ebrantil[Title/Abstract])) OR (urapidil, 2,3-(14)C2-labeled[Title/Abstract]))) OR ((Recombinant Human Brain Natriuretic Peptide[Title/Abstract]) OR (rhBNP[Title/Abstract]))) | 2,631 |
| 13 | (((((((((((((((((("Isosorbide Dinitrate"[Mesh]) OR (Isosorbide Dinitrate[Title/Abstract])) OR (Dinitrate, Isosorbide[Title/Abstract])) OR (Dilatrate[Title/Abstract])) OR (Iso Bid[Title/Abstract])) OR (Isodinit[Title/Abstract])) OR (Isoket[Title/Abstract])) OR (Sorbonit[Title/Abstract])) OR (Isomak R[Title/Abstract])) OR (Isordil[Title/Abstract])) OR (Isotrate[Title/Abstract])) OR (Nitrosorbide[Title/Abstract])) OR (Sorbitrate[Title/Abstract])) OR (Cardonit 40[Title/Abstract])) OR (Isoket Retard 120[Title/Abstract])) OR (((((((((((((((((((((((((((("Nitroglycerin"[Mesh]) OR (Nitroglycerin[Title/Abstract])) OR (Glyceryl Trinitrate[Title/Abstract])) OR (Trinitrate, Glyceryl[Title/Abstract])) OR (Nitrolan[Title/Abstract])) OR (Nitrospan[Title/Abstract])) OR (Nitrostat[Title/Abstract])) OR (Perlinganit[Title/Abstract])) OR (Nitrong[Title/Abstract])) OR (Susadrin[Title/Abstract])) OR (Sustac[Title/Abstract])) OR (Sustak[Title/Abstract])) OR (Sustonit[Title/Abstract])) OR (Transderm Nitro[Title/Abstract])) OR (Nitroglyn[Title/Abstract])) OR (Trinitrin[Title/Abstract])) OR (Trinitrolong[Title/Abstract])) OR (Anginine[Title/Abstract])) OR (Dynamite[Title/Abstract])) OR (Gilustenon[Title/Abstract])) OR (Nitrangin[Title/Abstract])) OR (Nitro Bid[Title/Abstract])) OR (Nitro Dur[Title/Abstract])) OR (Nitrocard[Title/Abstract])) OR (Nitroderm[Title/Abstract])) OR (Nitroderm TTS[Title/Abstract])) OR (Nitrol[Title/Abstract])) OR (Tridil[Title/Abstract]))) OR (((((((((((((((("Nitroprusside"[Mesh]) OR (Nitroprusside[Title/Abstract])) OR (Cyanonitrosylferrate[Title/Abstract])) OR (Nitroferricyanide[Title/Abstract])) OR (Nipruton[Title/Abstract])) OR (Nitriate[Title/Abstract])) OR (Nitropress[Title/Abstract])) OR (Nitroprusside, Disodium Salt, Dihydrate[Title/Abstract])) OR (Nipride[Title/Abstract])) OR (Naniprus[Title/Abstract])) OR (Nitroprussiat Fides[Title/Abstract])) OR (Sodium Nitroprusside[Title/Abstract])) OR (Nitroprusside, Sodium[Title/Abstract])) OR (Nitroprusside, Disodium Salt[Title/Abstract])) OR (Disodium Salt Nitroprusside[Title/Abstract])) OR (Ketostix[Title/Abstract]))) OR ((((((Urapidil[Title/Abstract]) OR (6-(3-(4-(o-methoxyphenyl)-1-piperazinyl)propylamino)-1,3-dimethyluracil[Title/Abstract])) OR (urapidil monohydrochloride[Title/Abstract])) OR (urapidil, 5-(14)C-labeled[Title/Abstract])) OR (Ebrantil[Title/Abstract])) OR (urapidil, 2,3-(14)C2-labeled[Title/Abstract]))) OR ((Recombinant Human Brain Natriuretic Peptide[Title/Abstract]) OR (rhBNP[Title/Abstract])) | 39,421 |
| 12 | (Recombinant Human Brain Natriuretic Peptide[Title/Abstract]) OR (rhBNP[Title/Abstract]) | 74 |
| 11 | (((((Urapidil[Title/Abstract]) OR (6-(3-(4-(o-methoxyphenyl)-1-piperazinyl)propylamino)-1,3-dimethyluracil[Title/Abstract])) OR (urapidil monohydrochloride[Title/Abstract])) OR (urapidil, 5-(14)C-labeled[Title/Abstract])) OR (Ebrantil[Title/Abstract])) OR (urapidil, 2,3-(14)C2-labeled[Title/Abstract]) | 732 |
| 10 | "urapidil" [Supplementary Concept] | 487 |
| 9 | ((((((((((((((("Nitroprusside"[Mesh]) OR (Nitroprusside[Title/Abstract])) OR (Cyanonitrosylferrate[Title/Abstract])) OR (Nitroferricyanide[Title/Abstract])) OR (Nipruton[Title/Abstract])) OR (Nitriate[Title/Abstract])) OR (Nitropress[Title/Abstract])) OR (Nitroprusside, Disodium Salt, Dihydrate[Title/Abstract])) OR (Nipride[Title/Abstract])) OR (Naniprus[Title/Abstract])) OR (Nitroprussiat Fides[Title/Abstract])) OR (Sodium Nitroprusside[Title/Abstract])) OR (Nitroprusside, Sodium[Title/Abstract])) OR (Nitroprusside, Disodium Salt[Title/Abstract])) OR (Disodium Salt Nitroprusside[Title/Abstract])) OR (Ketostix[Title/Abstract]) | 19,431 |
| 8 | "Nitroprusside"[Mesh] | 12,013 |
| 7 | ((((((((((((((((((((((((((("Nitroglycerin"[Mesh]) OR (Nitroglycerin[Title/Abstract])) OR (Glyceryl Trinitrate[Title/Abstract])) OR (Trinitrate, Glyceryl[Title/Abstract])) OR (Nitrolan[Title/Abstract])) OR (Nitrospan[Title/Abstract])) OR (Nitrostat[Title/Abstract])) OR (Perlinganit[Title/Abstract])) OR (Nitrong[Title/Abstract])) OR (Susadrin[Title/Abstract])) OR (Sustac[Title/Abstract])) OR (Sustak[Title/Abstract])) OR (Sustonit[Title/Abstract])) OR (Transderm Nitro[Title/Abstract])) OR (Nitroglyn[Title/Abstract])) OR (Trinitrin[Title/Abstract])) OR (Trinitrolong[Title/Abstract])) OR (Anginine[Title/Abstract])) OR (Dynamite[Title/Abstract])) OR (Gilustenon[Title/Abstract])) OR (Nitrangin[Title/Abstract])) OR (Nitro Bid[Title/Abstract])) OR (Nitro Dur[Title/Abstract])) OR (Nitrocard[Title/Abstract])) OR (Nitroderm[Title/Abstract])) OR (Nitroderm TTS[Title/Abstract])) OR (Nitrol[Title/Abstract])) OR (Tridil[Title/Abstract]) | 17,091 |
| 6 | (((((((((((((("Isosorbide Dinitrate"[Mesh]) OR (Isosorbide Dinitrate[Title/Abstract])) OR (Dinitrate, Isosorbide[Title/Abstract])) OR (Dilatrate[Title/Abstract])) OR (Iso Bid[Title/Abstract])) OR (Isodinit[Title/Abstract])) OR (Isoket[Title/Abstract])) OR (Sorbonit[Title/Abstract])) OR (Isomak R[Title/Abstract])) OR (Isordil[Title/Abstract])) OR (Isotrate[Title/Abstract])) OR (Nitrosorbide[Title/Abstract])) OR (Sorbitrate[Title/Abstract])) OR (Cardonit 40[Title/Abstract])) OR (Isoket Retard 120[Title/Abstract]) | 4,345 |
| 5 | "Isosorbide Dinitrate"[Mesh] | 3,543 |
| 3 | "Nitroglycerin"[Mesh] | 12,458 |
| 2 | ((((((Heart Failure [Title/Abstract]) OR (Cardiac Failure [Title/Abstract])) OR (Heart Decompensation [Title/Abstract])) OR (Decompensation, Heart [Title/Abstract])) OR (Myocardial Failure [Title/Abstract]))) OR ("Heart Failure"[Mesh]) | 238,380 |
| 1 | "Heart Failure"[Mesh] | 134,161 |

**Table S2** Characteristics of the included studies

| **Study ID** | **N (E/C)** | **Sex (M/F)** | **Age (years) (E/C)** | **I (E)** | **I (C)** | **Outcome** | **Scr(μmol/L)**  **(E/C)** | **Type of heart failure** | **Ejection fraction (%)**  **(E/C)** | **NT‐pro‐BNP (pg/mL)**  **(E/C)** |
| --- | --- | --- | --- | --- | --- | --- | --- | --- | --- | --- |
| Qian et al.,2019 | 196/198 | 194/200 | 66 ±10/ 66 ±9 | ISDN | PB | ① | - | congestive HF | 38±8/37±8 | - |
| Zamani et al.,2017 | 13/16 | 20/9 | 61 ±6.67/ 66.5±9.26 | ISDN | PB | ③ | - | HFpEF | 64.1±6/59.3 ±7.04 | - |
| Yang et al.,2017 | 89/91 | 105/75 | 77.5±5.3/ 76.9±11.3 | Urapidil | Nitroglycerin | ①②③④⑤ | 126.4 ±4.7/125.7 ±4.7 | acute HF | 37.1±4.2/36.2±4.2 | 7944.15±426.5/7946.73±379.98 |
| Xu et al.,2017 | 73/73 | 109/37 | 64.59±10.31/63.42±10.06 | rhBNP | Nitroglycerin | ③④⑤ | 245.85 ±7.24/224.51±6.37 | / | 32.3±6.7/31.9±6.5 | - |
| Wang et al.,2017 | 30/28 | 33/25 | 76.10±6.73/75.67±6.42 | Urapidil | Nitroglycerin | ②③④⑤ | 101.33±5.01/106.10±4.51 | acute HF | 47.86±6.27/46.86±5.87 | 9396.03±484.31/9140.13±311.21 |
| He et al.,2017 | 107/115 | 107/115 | 74.55±7.23/74.71±6.99 | Urapidil | Nitroglycerin | ①②③④⑤ | 107.68±47.48/90.58±48.11 | acute HF | 50.94±8.69/49.79±10.95 | 6178.63±7822.19/6142.86±7575.71 |
| Yang et al.,2016 | 58/62 | 70/50 | 78.0±10.0/ 78.1±10.2 | Urapidil | Nitroglycerin | ①②③⑤ | - | acute decompensated HF | 41.9±4.2/39.7±5.2 | 9140.67±795.76/9267.85±456.6 |
| Xing et al.,2016 | 57/59 | 81/35 | 58.91 ± 9.81/58.64 ± 11.51 | rhBNP | Nitroglycerin | ①②③ | 101.35±12.25/99.92±12.16 | / | 44.95 ± 7.80/47.43 ± 7.20 | - |
| Yang et al.,2015 | 35/37 | 39/33 | 70.68 ± 9.34/71.53 ± 11.28 | Urapidil | Nitroglycerin | ①②④⑤ | 98.43±4.77/94.49±4.99 | / | 50.13±4.17/47.14±6.23 | 7541.62±987.43/7321.45±946.23 |
| Wang et al.,2014 | 26/24 | 38/12 | 62.73±15.43/59.88±10.49 | rhBNP | Nitroglycerin | ①②③④ | 81.39±15.24/76.29±10.43 | acute HF | 49.87±9.24/53.47±7.81 | - |
| CHOW et al.,2011 | 44/45 | 48/41 | 66.8±17.2/ 68.6±14.1 | rhBNP | Nitroglycerin | ①②③④⑤ | 114.92±35.36/114.92±35.36 | acute decompensated HF | 32/33 | 2085±943/2226±1294 |
| Zhao et al.,2010 | 27/28 | 28/27 | 67.8 ± 7.0/ 66.4 ± 9.3 | rhBNP | nitroprusside | ①②③④ | 109.4±26.6/106.8±20 | acute decompensated HF | 33.9±4.6/36.4±4.8 | - |
| Mentzer Jr et al.,2007 | 141/138 | 219/60 | 63.6 ± 10.5/ 64.1 ± 11.3 | rhBNP | PB | ①③④ | - | HFrEF | 29.7±7.5/30.1 ± 7.3 | - |
| Peacock IV et al.,2005 | 120/117 | 132/105 | 66±16/ 65±15 | rhBNP | PB | ①③ | - | Congestive HF | - | - |
| Witteles et al.,2015 | 39/36 | 48/27 | 74.9/ 75.5 | rhBNP | PB | ③④ | - | acute decompensated HF | - | - |
| Dorszewski et al.,1999 | 18/18 | / | 55.7 ± 2.4/ 55.3 ±3.3 | urapidil | PB | ① | - | chronic congestive HF | - | - |
| Leier et al.,1983 | 13/17 | / | / | ISDN | PB | ① | - | moderate-to-severe congestive HF | - | - |
| C M O'Connor, et al.,2011 | 3496/3511 | 4616/2391 | 67±14.81/ 67±14.81 | rhBNP | PB | ①②③ | - | acute decompensated HF | - | - |

Note: ①Mortality; ②Re-admission rate; ③cardiovascular adverse events; ④scr; ⑤NT‐pro‐BNP.

Abbreviations: C, control group; E, experimental group; F, female; I, intervention; M, male.

Table S3: GRADE assessment

1. GRADE assessment (direct evidence) for mortality NMA

| Comparison groups | | Direct evidence | | | | | | | | | | | | | | |
| --- | --- | --- | --- | --- | --- | --- | --- | --- | --- | --- | --- | --- | --- | --- | --- | --- |
| Arm 1 | Arm 2(Ref) | No. of study | No. of patients | I-square,% | MD | LCI | UCI | RoB | Inconsistency | Indirectness | Publication bias | Direct rating without imprecision | imprecision | Direct rating with imprecision | Direct is more precise than indirect? | Need to rate indirect? |
| ISDN | PB | 2 | 424 | 29.1 | 2.62 | 0.4 | 19.59 | Serious | No | No | Undetected | Mod | Serious | Low | NA | No |
| ISDN | Urapidil |  |  |  |  |  |  |  |  |  |  |  |  |  |  |  |
| ISDN | Nitroglycerin |  |  |  |  |  |  |  |  |  |  |  |  |  |  |  |
| ISDN | rhBNP |  |  |  |  |  |  |  |  |  |  |  |  |  |  |  |
| ISDN | nitroprusside |  |  |  |  |  |  |  |  |  |  |  |  |  |  |  |
| Urapidil | PB | 1 | 36 | NA | 5.75 | 0.43 | 243.0 | No | No | No | Undetected | Mod | Serious | Low | Yes | Yes |
| Urapidil | Nitroglycerin | 5 | 652 | 18.5 | 0.38 | 0.05 | 1.8 | Serious | No | No | Undetected | Mod | Serious | Low | Yes | Yes |
| Urapidil | rhBNP |  |  |  |  |  |  |  |  |  |  |  |  |  |  |  |
| Urapidil | nitroprusside |  |  |  |  |  |  |  |  |  |  |  |  |  |  |  |
| Nitroglycerin | PB |  |  |  |  |  |  |  |  |  |  |  |  |  |  |  |
| rhBNP | Nitroglycerin | 3 | 310 | 45.8 | 0.88 | 0.21 | 3.29 | No | No | No | Undetected | Mod | Serious | Low | Yes | Yes |
| Nitroglycerin | nitroprusside |  |  |  |  |  |  |  |  |  |  |  |  |  |  |  |
| rhBNP | PB | 4 | 7598 | - | 0.97 | 0.43 | 3.32 | Serious | No | No | Undetected | Mod | Serious | Low | Yes | Yes |
| rhBNP | nitroprusside | 1 | 55 | NA | 0.4 | 0.01 | 9.17 | No | No | No | Undetected | Mod | Serious | Low | NA | No |
| nitroprusside | PB |  |  |  |  |  |  |  |  |  |  |  |  |  |  |  |

2. GRADE assessment (direct evidence) for re-admission rate NMA

| Comparison groups | | Direct evidence | | | | | | | | | | | | | | |
| --- | --- | --- | --- | --- | --- | --- | --- | --- | --- | --- | --- | --- | --- | --- | --- | --- |
| Arm 1 | Arm 2(Ref) | No. of study | No. of patients | I-square,% | RR | LCI | UCI | RoB | Inconsistency | Indirectness | Publication bias | Direct rating without imprecision | imprecision | Direct rating with imprecision | Direct is more precise than indirect? | Need to rate indirect? |
| Urapidil | PB |  |  |  |  |  |  |  |  |  |  |  |  |  |  |  |
| Urapidil | Nitroglycerin | 4 | 430 | 0.0 | 0.71 | 0.32 | 1.26 | Serious | No | No | Undetected | Mod | Serious | Low | NA | No |
| Urapidil | rhBNP |  |  |  |  |  |  |  |  |  |  |  |  |  |  |  |
| Urapidil | nitroprusside |  |  |  |  |  |  |  |  |  |  |  |  |  |  |  |
| Nitroglycerin | PB |  |  |  |  |  |  |  |  |  |  |  |  |  |  |  |
| rhBNP | Nitroglycerin | 2 | 166 | 0.0 | 1.55 | 0.37 | 7.27 | No | No | No | Undetected | Mod | Serious | Low | NA | No |
| Nitroglycerin | nitroprusside |  |  |  |  |  |  |  |  |  |  |  |  |  |  |  |
| rhBNP | PB | 1 | 7007 | NA | 0.89 | 0.33 | 2.87 | No | No | No | Undetected | Mod | Serious | Low | NA | No |
| rhBNP | nitroprusside | 1 | 55 | NA | 0.16 | 0 | 1.37 | No | No | No | Undetected | Mod | Serious | Low | NA | No |
| nitroprusside | PB |  |  |  |  |  |  |  |  |  |  |  |  |  |  |  |

3. GRADE assessment (direct evidence) for incidence of cardiovascular adverse events NMA

| Comparison groups | | Direct evidence | | | | | | | | | | | | | | |
| --- | --- | --- | --- | --- | --- | --- | --- | --- | --- | --- | --- | --- | --- | --- | --- | --- |
| Arm 1 | Arm 2(Ref) | No. of study | No. of patients | I-square,% | RR | LCI | UCI | RoB | Inconsistency | Indirectness | Publication bias | Direct rating without imprecision | imprecision | Direct rating with imprecision | Direct is more precise than indirect? | Need to rate indirect? |
| Urapidil | Nitroglycerin | 3 | 400 | 0.0 | 0.43 | 0.17 | 0.98 | Serious | No | No | Undetected | Mod | Serious | Low | NA | No |
| Urapidil | rhBNP |  |  |  |  |  |  |  |  |  |  |  |  |  |  |  |
| Urapidil | nitroprusside |  |  |  |  |  |  |  |  |  |  |  |  |  |  |  |
| rhBNP | Nitroglycerin | 1 | 50 | NA | 2.93 | 0.41 | 31.47 | No | No | No | Undetected | Mod | Serious | Low | NA | No |
| Nitroglycerin | nitroprusside |  |  |  |  |  |  |  |  |  |  |  |  |  |  |  |
| rhBNP | nitroprusside | 1 | 55 | NA | 0.44 | 0.09 | 2.12 | No | No | No | Undetected | Mod | Serious | Low | NA | No |

4. GRADE assessment (direct evidence) for Scr NMA

| Comparison groups | | Direct evidence | | | | | | | | | | | | | | |
| --- | --- | --- | --- | --- | --- | --- | --- | --- | --- | --- | --- | --- | --- | --- | --- | --- |
| Arm 1 | Arm 2(Ref) | No. of study | No. of patients | I-square,% | MD | LCI | UCI | RoB | Inconsistency | Indirectness | Publication bias | Direct rating without imprecision | imprecision | Direct rating with imprecision | Direct is more precise than indirect? | Need to rate indirect? |
| Urapidil | Nitroglycerin | 4 | 512 | 97.0 | 0.43 | -8.36 | 9.18 | Serious | Serious | No | Undetected | Mod | Serious | Low | NA | No |
| Urapidil | rhBNP |  |  |  |  |  |  |  |  |  |  |  |  |  |  |  |
| Urapidil | nitroprusside |  |  |  |  |  |  |  |  |  |  |  |  |  |  |  |
| rhBNP | Nitroglycerin | 3 | 255 | 66.1 | 0.48 | -10.79 | 11.99 | Serious | Serious | No | Undetected | Mod | Serious | Low | NA | No |
| Nitroglycerin | nitroprusside |  |  |  |  |  |  |  |  |  |  |  |  |  |  |  |
| rhBNP | nitroprusside | 1 | 55 | NA | -3.13 | -23.86 | 17.43 | No | No | No | Undetected | Mod | Serious | Low | NA | No |

5. GRADE assessment (direct evidence) for NT-pro-BNP NMA

| Comparison groups | | Direct evidence | | | | | | | | | | | | | | |
| --- | --- | --- | --- | --- | --- | --- | --- | --- | --- | --- | --- | --- | --- | --- | --- | --- |
| Arm 1 | Arm 2(Ref) | No. of study | No. of patients | I-square,% | MD | LCI | UCI | RoB | Inconsistency | Indirectness | Publication bias | Direct rating without imprecision | imprecision | Direct rating with imprecision | Direct is more precise than indirect? | Need to rate indirect? |
| Urapidil | Nitroglycerin | 5 | 652 | 93.0 | -975.64 | -1558.32 | -320.14 | Serious | Serious | No | Undetected | Mod | Serious | Low | NA | No |
| Urapidil | rhBNP |  |  |  |  |  |  |  |  |  |  |  |  |  |  |  |
| rhBNP | Nitroglycerin | 1 | 89 | NA | -126.51 | -1457.46 | 1203.99 | Serious | Serious | No | Undetected | Mod | Serious | Low | NA | No |

1. GRADE assessment (indirect evidence) for mortality NMA

| Comparison groups | | Indirect evidence | | | | | | | | Network evidence | | | | | | | |
| --- | --- | --- | --- | --- | --- | --- | --- | --- | --- | --- | --- | --- | --- | --- | --- | --- | --- |
| Arm1 | Arm2(Ref) | RR | LCI | UCI | Lowest of C1 and C2 | Intransitivity | Indirect rating  without  imprecision | Imprecision | Indirect rating  with imprecision | MD | LCI | UCI | Incoherence,  P-value | Higher rating of  direct and indirect  without imprecision | Incoherence | NMA  imprecision | Final  network  rating |
| ISDN | PB |  |  |  |  |  |  |  |  | 2.62 | 0.4 | 19.59 | NA | Mod | No | Serious | Low |
| ISDN | Urapidil | 2.04 | 0.14 | 27.53 | Low | No | Low | Serious | V Low | 2.04 | 0.14 | 27.53 | NA | Low | No | Serious | V Low |
| ISDN | Nitroglycerin | 1.35 | 0.09 | 14.37 | Mod | No | Mod | Serious | Low | 1.35 | 0.09 | 14.37 | NA | Mod | No | Serious | Low |
| ISDN | rhBNP | 2.19 | 0.21 | 18.3 | Low | No | Low | Serious | V Low | 2.19 | 0.21 | 18.3 | NA | Low | No | Serious | V Low |
| ISDN | nitroprusside | 0.85 | 0.01 | 35.69 | Mod | No | Mod | Serious | Low | 0.85 | 0.01 | 35.69 | NA | Mod | No | Serious | Low |
| Urapidil | PB | 0.42 | 0.04 | 4.98 | Mod | No | Mod | Serious | Low | 1.29 | 0.23 | 8.25 | 0.15 | Mod | No | Serious | Low |
| Urapidil | Nitroglycerin | 4.64 | 0.17 | 273.0 | Mod | No | Mod | Serious | Low | 0.66 | 0.12 | 2.57 | 0.15 | Mod | No | Serious | Low |
| Urapidil | rhBNP | 1.07 | 0.17 | 5.52 | Mod | No | Mod | Serious | Low | 1.07 | 0.17 | 5.52 | NA | Mod | No | Serious | Low |
| Urapidil | nitroprusside | 0.41 | 0.01 | 14.61 | Mod | No | Mod | Serious | Low | 0.41 | 0.01 | 14.61 | NA | Mod | No | Serious | Low |
| Nitroglycerin | PB | 1.92 | 0.5 | 12.78 | Mod | No | Mod | Serious | Low | 1.92 | 0.5 | 12.78 | NA | Mod | No | Serious | Low |
| rhBNP | Nitroglycerin | 0.07 | 0.00 | 1.89 | Mod | No | Mod | Serious | Low | 0.62 | 0.15 | 2.21 | 0.16 | Mod | No | Serious | Low |
| Nitroglycerin | nitroprusside | 0.64 | 0.01 | 21.2 | Mod | No | Mod | Serious | Low | 0.64 | 0.01 | 21.2 | NA | Mod | No | Serious | Low |
| rhBNP | PB | 13.3 | 0.45 | 997.0 | Mod | No | Mod | Serious | Low | 1.16 | 0.52 | 4.26 | 0.15 | Mod | No | Serious | Low |
| rhBNP | nitroprusside |  |  |  |  |  |  |  |  | 0.4 | 0.01 | 9.17 | NA | Mod | No | Serious | Low |
| nitroprusside | PB | 3.11 | 0.12 | 181.36 | Mod | No | Mod | Serious | Low | 3.11 | 0.12 | 181.36 | NA | Mod | No | Serious | Low |

2. GRADE assessment (indirect evidence) for re-admission rate NMA

| Comparison groups | | Indirect evidence | | | | | | | | Network evidence | | | | | | | |
| --- | --- | --- | --- | --- | --- | --- | --- | --- | --- | --- | --- | --- | --- | --- | --- | --- | --- |
| Arm1 | Arm2(Ref) | RR | LCI | UCI | Lowest of C1 and C2 | Intransitivity | Indirect rating  without  imprecision | Imprecision | Indirect rating  with imprecision | MD | LCI | UCI | Incoherence,  P-value | Higher rating of  direct and indirect  without imprecision | Incoherence | NMA  imprecision | Final  network  rating |
| Urapidil | PB | 0.44 | 0.06 | 2.68 | Mod | No | Mod | Serious | Low | 0.44 | 0.06 | 2.68 | NA | Mod | No | Serious | Low |
| Urapidil | Nitroglycerin |  |  |  |  |  |  |  |  | 0.71 | 0.32 | 1.26 | NA | Mod | No | Serious | Low |
| Urapidil | rhBNP | 0.45 | 0.08 | 2.07 | Low | No | Low | Serious | V Low | 0.45 | 0.08 | 2.07 | NA | Low | No | Serious | V Low |
| Urapidil | nitroprusside | 0.07 | 0 | 1.02 | Low | No | Low | Serious | V Low | 0.07 | 0 | 1.02 | NA | Low | No | Serious | V Low |
| PB | Nitroglycerin | 1.58 | 0.27 | 10.07 | Mod | No | Mod | Serious | Low | 1.58 | 0.27 | 10.07 | NA | Mod | No | Serious | Low |
| rhBNP | Nitroglycerin |  |  |  |  |  |  |  |  | 1.55 | 0.37 | 7.27 | NA | Mod | No | Serious | Low |
| nitroprusside | Nitroglycerin | 10.16 | 0.72 | 460.51 | Mod | No | Mod | Serious | Low | 10.16 | 0.72 | 460.51 | NA | Mod | No | Serious | Low |
| rhBNP | PB |  |  |  |  |  |  |  |  | 0.89 | 0.33 | 2.87 | NA | Mod | No | Serious | Low |
| rhBNP | nitroprusside |  |  |  |  |  |  |  |  | 0.16 | 0 | 1.37 | NA | Mod | No | Serious | Low |
| PB | nitroprusside | 0.16 | 0 | 1.76 | Mod | No | Mod | Serious | Low | 0.16 | 0 | 1.76 | NA | Mod | No | Serious | Low |

3. GRADE assessment (indirect evidence) for incidence of cardiovascular adverse events NMA

| Comparison groups | | Indirect evidence | | | | | | | | Network evidence | | | | | | | |
| --- | --- | --- | --- | --- | --- | --- | --- | --- | --- | --- | --- | --- | --- | --- | --- | --- | --- |
| Arm1 | Arm2(Ref) | RR | LCI | UCI | Lowest of C1 and C2 | Intransitivity | Indirect rating  without  imprecision | Imprecision | Indirect rating  with imprecision | MD | LCI | UCI | Incoherence,  P-value | Higher rating of  direct and indirect  without imprecision | Incoherence | NMA  imprecision | Final  network  rating |
| Urapidil | Nitroglycerin |  |  |  |  |  |  |  |  | 0.43 | 0.17 | 0.98 | NA | Mod | No | Serious | Low |
| Urapidil | rhBNP | 0.15 | 0.01 | 1.21 | Mod | No | Mod | Serious | Low | 0.15 | 0.01 | 1.21 | NA | Mod | No | Serious | Low |
| Urapidil | nitroprusside | 0.06 | 0 | 0.9 | Low | No | Low | Serious | V Low | 0.06 | 0 | 0.9 | NA | Low | No | Serious | V Low |
| rhBNP | Nitroglycerin |  |  |  |  |  |  |  |  | 2.93 | 0.41 | 31.47 | NA | Mod | No | Serious | Low |
| nitroprusside | Nitroglycerin | 6.81 | 0.53 | 112.95 | Mod | No | Mod | Serious | Low | 6.81 | 0.53 | 112.95 | NA | Mod | No | Serious | Low |
| rhBNP | nitroprusside |  |  |  |  |  |  |  |  | 0.44 | 0.09 | 2.12 | NA | Mod | No | Serious | Low |

4. GRADE assessment (indirect evidence) for Scr NMA

| Comparison groups | | Indirect evidence | | | | | | | | Network evidence | | | | | | | |
| --- | --- | --- | --- | --- | --- | --- | --- | --- | --- | --- | --- | --- | --- | --- | --- | --- | --- |
| Arm1 | Arm2(Ref) | MD | LCI | UCI | Lowest of C1 and C2 | Intransitivity | Indirect rating  without  imprecision | Imprecision | Indirect rating  with imprecision | MD | LCI | UCI | Incoherence,  P-value | Higher rating of  direct and indirect  without imprecision | Incoherence | NMA  imprecision | **Final**  **network**  **rating** |
| Urapidil | Nitroglycerin |  |  |  |  |  |  |  |  | 0.43 | -8.36 | 9.18 | NA | Mod | No | Serious | Low |
| Urapidil | rhBNP | -0.06 | -14.63 | 14.19 | Mod | No | Mod | Serious | Low | -0.06 | -14.63 | 14.19 | NA | Mod | No | Serious | Low |
| Urapidil | nitroprusside | -3.19 | -28.46 | 21.75 | Low | No | Low | Serious | V Low | -3.19 | -28.46 | 21.75 | NA | Low | No | Serious | V Low |
| rhBNP | Nitroglycerin |  |  |  |  |  |  |  |  | 0.48 | -10.79 | 11.99 | NA | Mod | No | Serious | Low |
| nitroprusside | Nitroglycerin | 3.62 | -19.87 | 27.37 | Mod | No | Mod | Serious | Low | 3.62 | -19.87 | 27.37 | NA | Mod | No | Serious | Low |
| rhBNP | nitroprusside |  |  |  |  |  |  |  |  | -3.13 | -23.86 | 17.43 | NA | Mod | No | Serious | Low |

5. GRADE assessment (indirect evidence) for NT-pro-BNP NMA

| **Comparison groups** | | **Indirect evidence** | | | | | | | | **Network evidence** | | | | | | | |
| --- | --- | --- | --- | --- | --- | --- | --- | --- | --- | --- | --- | --- | --- | --- | --- | --- | --- |
| Arm1 | Arm2(Ref) | **MD** | **LCI** | **UCI** | **Lowest of C1 and C2** | **Intransitivity** | **Indirect rating**  **without**  **imprecision** | **Imprecision** | **Indirect rating**  **with imprecision** | **MD** | **LCI** | **UCI** | **Incoherence,**  **P-value** | **Higher rating of**  **direct and indirect**  **without imprecision** | **Incoherence** | **NMA**  **imprecision** | **Final**  **network**  **rating** |
| Urapidil | Nitroglycerin |  |  |  |  |  |  |  |  | -975.64 | -1558.32 | -320.14 | NA | Mod | No | Serious | Low |
| Urapidil | rhBNP | -848.4 | -2277.02 | 656.78 | Low | No | Low | Serious | V Low | -848.4 | -2277.02 | 656.78 | NA | Low | No | Serious | V Low |
| rhBNP | Nitroglycerin |  |  |  |  |  |  |  |  | -126.51 | -1457.46 | 1203.99 | NA | Mod | No | Serious | Low |

**Fig. S1** Risk of bias graph

**Fig. S2** Funnel plots of outcomes

(a) Mortality, (b) re-admission rate, (c) incidence of cardiovascular adverse events, (d) sCr, (e) NT-pro-BNP
